# Tumour tissue-associated microbiome differences between colonic adenoma and carcinoma revealed by 5R 16S rRNA sequencing of formalin-fixed paraffin-embedded tissues: a case–control study

**DOI:** 10.3389/fmicb.2026.1880194

**Published:** 2026-07-10

**Authors:** Hanyang Yi, Wanneng He, Zijia Ma, Xianfeng Qiu, Lihua Ren, Xuyang Liang

**Affiliations:** 1 Department of Gastroenterology, The Affiliated Lianyungang Hospital of Xuzhou Medical University/The First People's Hospital of Lianyungang, Lianyungang, China; 2 Department of Pathology, The Affiliated Lianyungang Hospital of Xuzhou Medical University/The First People's Hospital of Lianyungang, Lianyungang, China; 3 Department of Gastroenterology, Lianyungang Clinical College of Nanjing Medical University/The First People's Hospital of Lianyungang, Lianyungang, China; 4 Department of Gastroenterology, Zhongda Hospital, Southeast University, Nanjing, China

**Keywords:** 5R 16S rRNA, *Alistipes finegoldii*, *Bacteroides caccae*, colon cancer, colonic adenoma, formalin-fixed paraffin-embedded, *Prevotella intermedia*, tumour microbiota

## Abstract

Tumour-associated microbiota directly colonise lesion sites and interact with the local immune microenvironment, yet their compositional shifts during the adenoma-to-cancer transition remain poorly characterised, particularly in archival tissue. Using five-region 16S ribosomal RNA (5R 16S rRNA) sequencing with a four-category contamination control framework, we compared tumour-associated microbiomes in formalin-fixed paraffin-embedded (FFPE) tissues from 25 colonic tubular adenoma and 25 colon cancer patients in a case–control design. Alpha diversity was significantly reduced in the colon cancer group across three indices (Shannon, Simpson, and Pielou’s evenness), reflecting shifts in community evenness rather than overall species loss. Beta diversity analysis, however, revealed no significant differences in global community composition between groups (ANOSIM: *R* = 0.026, *p* = 0.106; PERMANOVA: *R*^2^ = 0.024, *p* = 0.137), indicating that observed compositional differences are taxon-specific rather than community-wide. Across four independent statistical methods (ANCOM-BC2, LEfSe, Mann–Whitney U test, and Fisher’s exact test), *Bacteroides caccae* and *Prevotella intermedia* were consistently enriched in colon cancer tissue, suggesting compositional shifts in the tumour-associated microbiome that may be associated with colorectal carcinogenesis. Within the colon cancer group, *Alistipes finegoldii* abundance showed a negative correlation with tumour diameter across all colon cancer samples (Spearman *r* = −0.520, *p* = 0.008; *n* = 25, including 20 samples at the detection limit as tied ranks; only 5 samples had detectable abundance); given the very limited number of detectable samples, this finding should be regarded as strictly exploratory and requires prospective validation. These findings demonstrate that tumour-associated microbiome profiling in archival FFPE tissue is feasible under a rigorous contamination control framework, and provide preliminary evidence for taxon-specific compositional differences during colonic adenoma-to-carcinoma progression. The identified candidate taxa warrant prospective validation in larger cohorts and mechanistic investigation to clarify their roles in colon carcinogenesis.

## 1 Introduction

Colorectal cancer (CRC) is the third most common malignant tumour worldwide in incidence and the second leading cause of cancer-related deaths (Bray et al., 2024; Siegel et al., 2024). Colorectal adenomas (CRA) are widely recognised as the primary precancerous lesions of CRC (Strum, 2016; Wang et al., 2025). Consequently, early diagnosis and intervention at the adenoma stage are of significant clinical importance for halting the “adenoma-to-cancer” progression and reducing the burden of CRC (Bray et al., 2024; Jiang et al., 2024).

Recent studies have demonstrated that gut microbiota dysbiosis plays a role in the development and progression of CRC (Wang et al., 2025; Wong and Yu, 2023; Wang et al., 2023; Wong and Yu, 2019; Garrett, 2015). Specific bacterial species (e.g., *Fusobacterium nucleatum* and *Peptostreptococcus stomatis*) are enriched in CRC tissues (Nejman et al., 2020; Huang et al., 2024). However, existing studies have largely focused on faecal samples or normal mucosa, which reflect only “transient” microorganisms within the intestinal lumen; the association between tumour-colonising microorganisms and the adenoma and carcinoma stages remains unclear (Shakhpazyan et al., 2025). In contrast, tumour-associated microbiota directly colonise the lesion site, interact more closely with the host’s local immune and metabolic microenvironment, and are less susceptible to interference from short-term dietary and environmental fluctuations (Nejman et al., 2020; Galeano Niño et al., 2022), making them an active area of investigation in tumour microbiome research.

However, the findings of current research on the tumour-associated microbiome in CRC remain controversial, primarily due to the heterogeneity in anatomical location and pathological subtype control across existing studies. On the one hand, the colonisation of gut microbiota exhibits significant spatial and anatomical heterogeneity, with different anatomical locations within the intestine having a marked influence on the composition of the gut microbiota (Dekker et al., 2019; Shi et al., 2025). On the other hand, colorectal adenomas are highly complex histologically (for example, the malignant potential of villous adenomas differs markedly from that of tubular adenomas; Lo et al., 2022). Analysing samples from different anatomical sites or complex pathological subtypes together may mask true microbial differences (Liu et al., 2025; Flemer et al., 2017).

In studies of the tumour-associated microbiome, fresh-frozen (FF) tissue is generally regarded as the gold standard for extracting high-abundance microbial DNA. However, its acquisition is highly dependent on stringent intraoperative liquid nitrogen rapid-freezing conditions, which are difficult to implement in long-term, large-scale retrospective clinical cohort studies. In contrast, formalin-fixed paraffin-embedded (FFPE) tissue is the most common form of archival specimen in clinical pathology. Not only is the volume of such samples vast, but they can also be perfectly matched with patients’ complete long-term follow-up data and pathological characteristics, and are increasingly being used in clinical research (Chang et al., 2023). Subsequent validation studies have confirmed that FFPE-based 16S profiling yields reproducible core microbial signals: Borgognone et al. (2021) demonstrated >90% concordance in core taxa between FFPE and fresh-frozen tissue; An et al. (2024) confirmed feasibility with appropriate decontamination in intratumoral microbiome studies; and Fongmanee et al. (2025) validated FFPE utility for mucosa-associated microbiota analysis in a retrospective Asian cohort. However, the inherent cross-linking and degradation, as well as DNA fragmentation and an extremely low microbial load in FFPE samples, mean that conventional 16S amplification often faces a high risk of background contamination and a high rate of false negatives (Cruz-Flores et al., 2022).

This study employed five-region 16S ribosomal RNA (5R 16S rRNA) sequencing technology to conduct a comparative analysis of the tumour-associated microbiome in rigorously selected archived FFPE adenoma and colon cancer tissues. The aim was to explore the characteristic differences in the microbiome along the adenoma-to-cancer transition, assess the association between these microbial differences and tumour clinical characteristics, and provide preliminary microbiome-based evidence for compositional differences in the tumour-associated microbial community during colon carcinogenesis.

## 2 Materials and methods

This study adhered to the ethical principles of the Declaration of Helsinki and was approved by the Institutional Review Board (IRB) of The First People’s Hospital of Lianyungang (approval No. KY-20251027003-01). As this was a retrospective analysis, the study was exempt from the requirement for informed consent from patients. Patients who underwent colonoscopy or surgical resection at The First People’s Hospital of Lianyungang between February 2025 and February 2026 were included. Through a systematic search of the Department of Pathology’s FFPE specimen repository and clinical records, a total of 25 eligible patients with colonic adenomas and 25 patients with colon cancer were ultimately selected, meeting the inclusion and exclusion criteria. All patients were from the same region and had similar dietary habits.

### 2.1 Study population

#### 2.1.1 Patients with colon cancer

Inclusion criteria: (1) Pathologically confirmed colorectal adenocarcinoma (stage pT3 or higher) located in the left or right half of the colon.

Exclusion criteria: (1) Poorly differentiated adenocarcinoma, mucinous adenocarcinoma, signet-ring cell carcinoma, or rectal tumours; (2) specimens showing significant necrosis; (3) previous treatment with radiotherapy or chemotherapy; (4) use of antibiotics within 1 month before surgery or proton pump inhibitors within 2 weeks before colonoscopy.

#### 2.1.2 Patients with colonic adenomas

Inclusion criteria: (1) Pathologically confirmed tubular adenoma with low-grade dysplasia, located in the left or right half of the colon. (2) specimens collected via endoscopic mucosal resection (EMR), endoscopic submucosal dissection (ESD), or polypectomy snare resection to ensure adequate tissue volume.

Exclusion criteria: (1) Villous or mixed-type adenomas; (2) rectal adenomas; (3) specimens with substandard DNA quality.

### 2.2 Methods

#### 2.2.1 Sample identification and pre-processing

All samples were independently identified by two senior pathologists using haematoxylin and eosin (HE)-stained sections examined under a light microscope in accordance with the 2019 World Health Organisation (WHO) Classification of Tumours of the Digestive System (Nagtegaal et al., 2020). Patient demographic data (age, sex, BMI) and clinical characteristics (tumour location, size, CEA, CA19-9, etc.) were collected concurrently. FFPE sections were cut to a thickness of 20 μm, with 6–10 consecutive sections taken from each sample. These were placed in sterile, enzyme-free centrifuge tubes and stored refrigerated, then transported via cold chain to the laboratory for analysis within 48 h of collection.

#### 2.2.2 Contamination control and negative control design

As FFPE samples are characterised by low biomass, this study established a system comprising four types of negative contamination controls covering the entire process from sampling to DNA extraction and PCR amplification. All control samples were sequenced in the same batch as the test samples. The specific design is as follows:

Sampling Negative Controls (*n* = 4, comprising two batches of two samples each): These included an ambient air control (a cotton swab dipped in distilled water was used to wipe the microtome blade and placed in a tube) and a paraffin blank control (pure paraffin was excised from the non-tissue margin of a paraffin block). Two samples were from the main experimental batch and two from the pilot batch.

DNA extraction negative controls (*n* = 5): No tissue samples were added; extraction was performed simultaneously using only the same lysis buffer to monitor background contamination in the extraction reagents.

PCR amplification no-template controls (*n* = 5): Sterile water was used in place of the DNA template for simultaneous amplification to monitor contamination of the primers and the amplification environment.

The main experimental batch—comprising all 50 tissue samples, 2 sampling negative controls, 5 DNA extraction controls, and 5 PCR no-template controls (*n* = 62 total)—was processed and sequenced within a single sequencing run. Two additional sampling negative controls from the pilot run were included as supplementary contamination references.

A four-stage contamination removal pipeline was applied sequentially to all samples. In Stage 1, low-depth libraries with fewer than 1,000 total reads were excluded prior to downstream filtering. In Stage 2, ASVs with a relative abundance below 10^−4^ across all samples were removed. In Stage 3, ASVs detected in more than 7.5% of negative control samples were systematically excluded; this threshold was pre-specified prior to sequencing and applied uniformly without post-hoc adjustment. In Stage 4, retained ASVs were cross-referenced against the Salter et al. (2014) reagent contaminant database, and taxa identified as known reagent contaminants were removed. This procedure employs the conservative filtering criteria recommended for low-biomass microbiome studies (Eisenhofer et al., 2019). As a retrospective sensitivity analysis, the decontam algorithm (prevalence method, *p* = 0.1; Davis et al., 2018) was applied to verify the biological authenticity of differentially abundant taxa (Supplementary Table S4).

#### 2.2.3 5R 16S rRNA gene amplification and sequencing

After adding lysis buffer to the tissue samples, homogenisation was performed using a Tissuelyser-48 tissue homogeniser (60 Hz). Total DNA was extracted using the CTAB method and quantified using a NanoDrop 2000 and agarose gel electrophoresis. Amplification was carried out using the 5R amplification strategy based on the Short-fragment Multiple-region Integrated Framework (SMURF) algorithm (Fuks et al., 2018). Five pairs of specific primers were used to simultaneously target the five hypervariable regions (V2, V3, V5, V6 and V8) of the bacterial 16S rRNA gene. After purification, the products were sequenced using paired-end sequencing (2 × 250 bp) on the Illumina NovaSeq 6000 platform.

#### 2.2.4 Statistical and bioinformatics analyses

Alpha diversity indices (Chao1, Shannon, Simpson, Pielou’s evenness) were calculated; group differences in alpha diversity were assessed using the Wilcoxon rank-sum test. Beta diversity was assessed using Analysis of Similarity (ANOSIM) and permutational multivariate analysis of variance (PERMANOVA) (999 permutations), both based on the Bray–Curtis dissimilarity matrix, implemented in the R package vegan. A clustering heatmap was constructed to visualise the overall distribution pattern of species-level abundance between groups. Differentially abundant taxa were identified using the Linear Discriminant Analysis Effect Size (LEfSe) platform (LDA threshold > 3.0) and the Analysis of Compositions of Microbiomes with Bias Correction 2 (ANCOM-BC2) algorithm (Lin and Peddada, 2024); feature importance analysis for random forests was performed using the R package randomForest; Mann–Whitney U tests, Fisher’s exact tests and Spearman’s rank correlation analyses were performed using SPSS 27.0 (IBM, Armonk, NY); Binary logistic regression was performed using Firth’s penalised maximum likelihood method (R package logistf, version 1.26.0; Heinze and Schemper, 2002). For each taxon, a primary model adjusted for sex and a sensitivity model additionally adjusted for age and BMI were pre-specified. Data visualisation was carried out using GraphPad Prism 9.0 and the R package ggplot2. A *p*-value < 0.05 in two-tailed tests was considered statistically significant.

#### 2.2.5 Differential taxa identification and clinical correlation analysis

A two-step approach was employed to identify differentially abundant taxa. First, ANCOM-BC2 (applied with group as the sole predictor variable and Benjamini–Hochberg FDR correction; Lin and Peddada, 2024) was used to screen for differentially abundant taxa, with Fisher’s exact test additionally applied to assess detection rate differences. Next, Mann–Whitney U tests were used to compare relative abundance distributions and Fisher’s exact test to compare detection rates between groups. Finally, Spearman’s rank correlation analysis was applied to assess associations between core differential taxa and clinical characteristics (tumour diameter, CEA, Ki67); results are presented as scatter plots with 95% confidence intervals.

## 3 Results

### 3.1 Clinical and pathological characteristics

This study included 25 patients with colon cancer and 25 patients with colonic adenomas; their clinical baseline characteristics are shown in Table 1. There were no significant differences between the two groups in gender, age, BMI, comorbidities (hypertension, diabetes) or anatomical location of the tumour (left/right colon) (all *p >* 0.05). The mean tumour diameter and serum CEA levels in the colon cancer group were higher than those in the adenoma group (both *p <* 0.001). Specific clinical and pathological characteristics of the colon cancer group (staging, MMR status, Ki67 index, etc.) are presented in Table 2.

**Table 1:** Baseline clinical characteristics of participants.

Characteristics	(Colonic adenoma patients, *n* = 25)	(Colon cancer patients, *n* = 25)	*p* value
Age	59 ± 10	62 ± 11	0.233^a^
Sex			0.145^c^
Female	12 (48%)	7 (28%)	
Male	13 (52%)	18 (72%)	
BMI (kg/m^2^)	23.36 ± 3.42	23.08 ± 2.67	0.751^a^
Tumour size (cm)	1.62 ± 0.69	4.48 ± 1.63	<0.001^a^
Tumour markers			
CEA(ng/mL; *n* = 41)	1.69 (1.44, 2.62)	4.78 (2.59, 7.86)	<0.001^b^
CA19-9 (ng/mL; *n* = 40)	13.90 (7.67, 17.60)	14.5 (8.25, 30.70)	0.551^b^
Comorbidity			
**Hypertension**			0.774^c^
No	15 (60%)	14 (56%)	
Yes	10 (40%)	11 (44%)	
Diabetes mellitus			0.185^c^
No	21 (84%)	17 (68%)	
Yes	4 (16%)	8 (32%)	
Tumour site			0.564^c^
Site 1(Left-sided colon)	16 (64%)	15 (60%)	
Site 2(Right-sided colon)	9 (36%)	10 (40%)	

CEA data available for 41 patients; CA19-9 data available for 40 patients. Missing data were not significantly different between groups.

a Independent samples *t*-test.

b Mann–Whitney U test.

c Chi-squared test.

**Table 2:** Pathological characteristics of the colon cancer group.

Pathological characteristics of the colon cancer group	N (%)
Lymph node stage (pN)	
PN0	14 (56%)
PN1	9 (36%)
PN2	2 (8%)
MMR Status	
pMMR	23 (92%)
MSI-H	2 (8%)
Perineural invasion	16 (64%)
Lymphovascular invasion	10 (40%)
Tumour budding	
Low-grade	6 (24%)
Intermediate-grade	14 (56%)
High-grade	5 (20%)
Ki67%	80 (70, 83)^a^

MMR, mismatch repair; pMMR, proficient mismatch repair; MSI-H, microsatellite instability-high.

a Median (interquartile range).

### 3.2 Assessment of sequencing data quality control and background noise reduction

Given the low biomass characteristic of FFPE samples, this study verified the sequencing signals of four types of negative contamination controls. Although trace amounts of gut microbiota signals were detected in the sampling negative control, the key point is that the relative abundance and absolute sequence counts of core gut bacteria in the formal tumour tissue samples were several orders of magnitude higher than those in the corresponding negative controls. Raw sequencing of the 50 tissue samples yielded a mean of 259,516 ± 29,762 reads per sample. After paired-end merging and quality trimming (Q > 20), a mean of 244,383 ± 28,279 high-quality reads per sample were retained (mean retention rate: 94.2%; mean Q30 rate: 92.4%, range 91.5–93.2%; Supplementary Table S2). Subsequent rarefaction curve analysis indicated that all samples had reached sequencing saturation (Supplementary Figure S1). The four-stage contamination removal pipeline progressively reduced the ASV dataset: after Stage 2 relative-abundance filtering, 5,305 ASVs were retained for further processing. Stage 3 filtering (7.5% negative-control prevalence threshold) reduced these to 4,247 ASVs (19.9% reduction; Supplementary Table S3). Stage 4 cross-referencing against the Salter et al. (2014) reagent contaminant database further reduced the dataset to 2,970 ASVs, which constituted the final feature table used in all downstream statistical analyses. Retrospective decontam analysis confirmed that none of the four differentially abundant taxa were flagged as potential contaminants (Supplementary Table S4).

### 3.3 Microbial *α*-diversity

**Figure 1:**
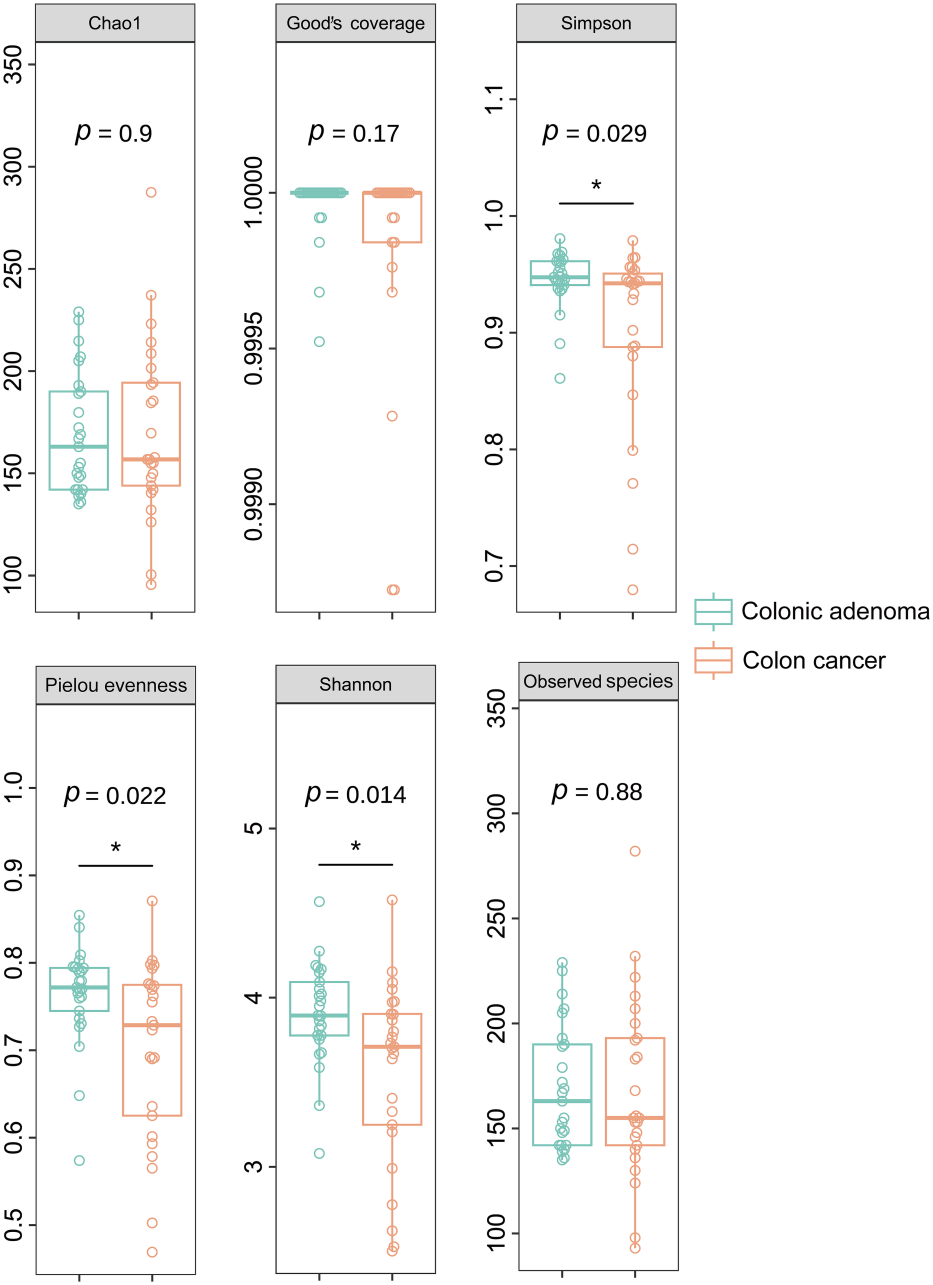
Alpha diversity of tumour tissue-associated microbiota. Alpha diversity indices of tumour tissue-associated microbiota in colonic adenoma and colon cancer. Box plots display Shannon, Simpson, Pielou’s evenness, Chao1, observed species, and Good’s coverage indices. Statistical differences between groups were assessed by Wilcoxon rank-sum test. *p < 0.05.

The richness and evenness of the microbiota associated with tumour tissue were assessed by calculating α-diversity indices (Figure 1). Compared with the adenoma group, both the richness and evenness of tumour-associated microbiota were reduced in the colon cancer group. In the colon cancer group, the Shannon index (*p =* 0.014), Simpson’s index (*p =* 0.029) and Pielou’s evenness index (*p =* 0.022) were all lower than those in the colonic adenoma group; however, no significant differences were observed between the two groups in the indices reflecting absolute species richness, such as the Chao1, observed species, and Good’s coverage indices (*p >* 0.05).

### 3.4 Microbial community structure and *β*-diversity

**Figure 2:**
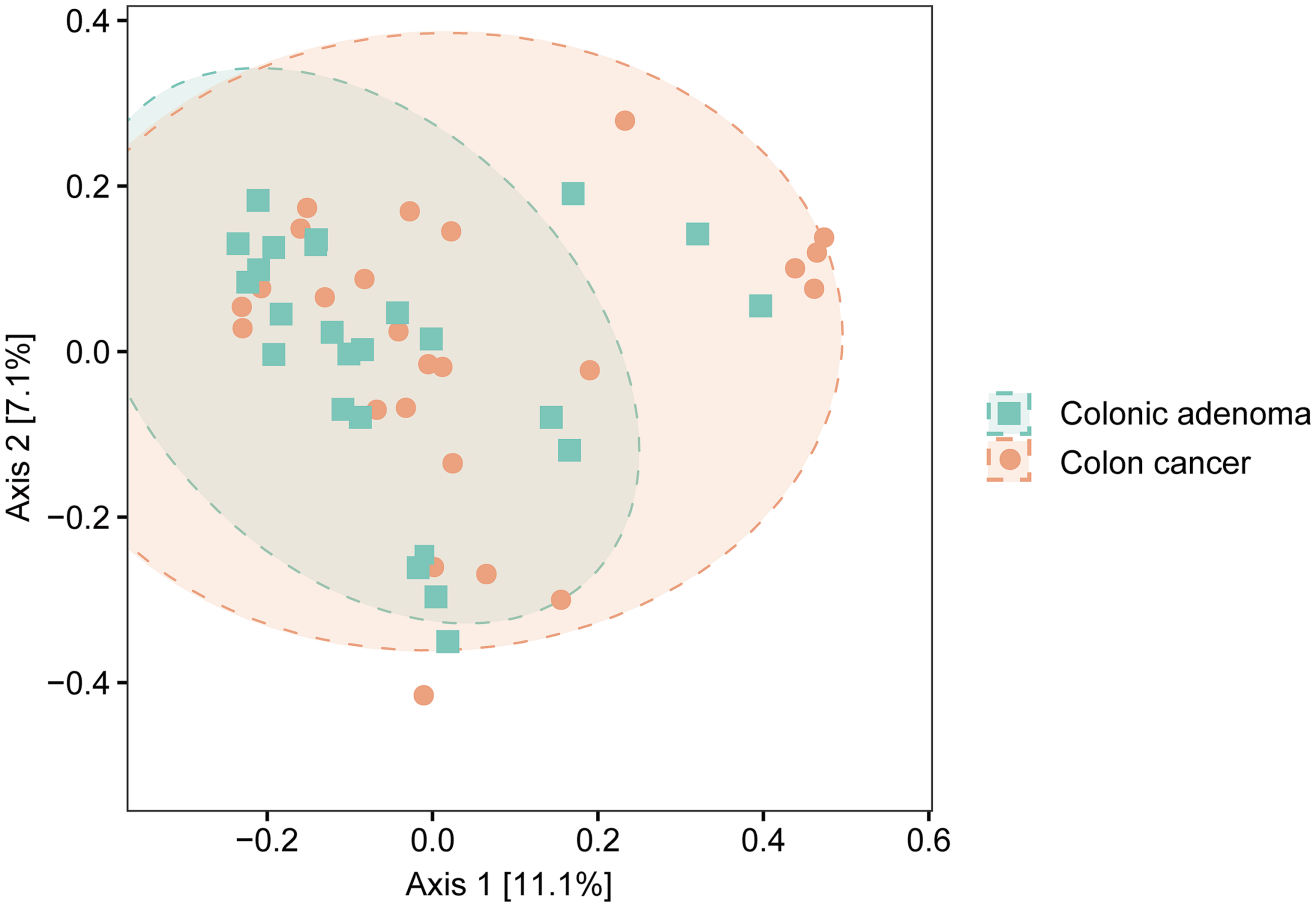
Beta diversity based on Bray–Curtis dissimilarity (PCoA). Principal Coordinates Analysis (PCoA) based on Bray–Curtis dissimilarity matrix. Teal squares represent the colonic adenoma group; orange circles represent the colon cancer group. ANOSIM: *R* = 0.026, *p* = 0.106; PERMANOVA: *R*^2^ = 0.024, *p* = 0.137. Neither test reached statistical significance, indicating substantial overlap in overall community structure between groups.

Principal Coordinates Analysis (PCoA) based on the Bray–Curtis dissimilarity matrix was performed to evaluate the overall microbial community structure between the two groups (Figure 2). The PCoA plot demonstrated a substantial spatial overlap between the colonic adenoma and colon cancer clusters along both principal axes (Axis 1: 11.1%; Axis 2: 7.1%). Formal statistical testing confirmed that the global compositional differences between the groups did not reach statistical significance, as verified by both the ANOSIM test (*R* = 0.026, *p* = 0.106) and the PERMANOVA analysis (*R*^2^ = 0.024, *p* = 0.137).

### 3.5 Microbial composition associated with tumour tissue

**Figure 3:**
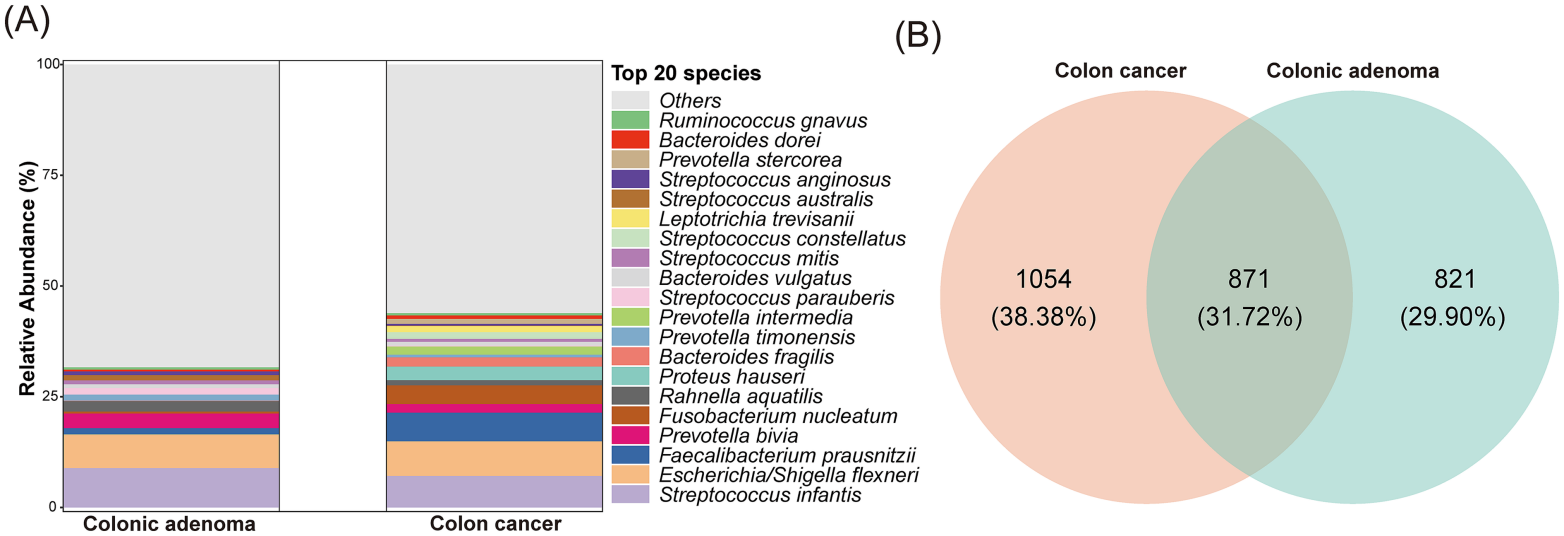
Tumour tissue-associated microbial community composition. Intratumoural microbial community composition. **(A)** Stacked bar chart showing relative abundance of the top 20 species in colonic adenoma and colon cancer groups. **(B)** Venn diagram illustrating the number and proportion of shared and group-specific ASVs between groups.

After filtering out four categories of negative contamination controls, we analysed the community composition at the species level (Figure 3A). The stacked bar chart shows that the 20 most abundant species account for approximately 30 to 45% of the entire community, with the remaining unidentified microorganisms classified as “Others.” Core gut microbiota, such as *Bacteroides vulgatus* and *Bacteroides fragilis*, were consistently detected in both sample groups. Compared with the adenoma group, the detection rates of the aforementioned gut-associated bacterial species were generally higher in the colon cancer group. *Fusobacterium nucleatum* and *Prevotella intermedia* were detected in the colon cancer group and exhibited an enrichment trend. Analysis using a Venn diagram (Figure 3B) further indicated that approximately one-third of the amplicon sequence variants (ASVs) in each group were shared taxa, while each group possessed approximately one-third of unique ASVs.

### 3.6 Identification of microbial differential taxa

**Figure 4:**
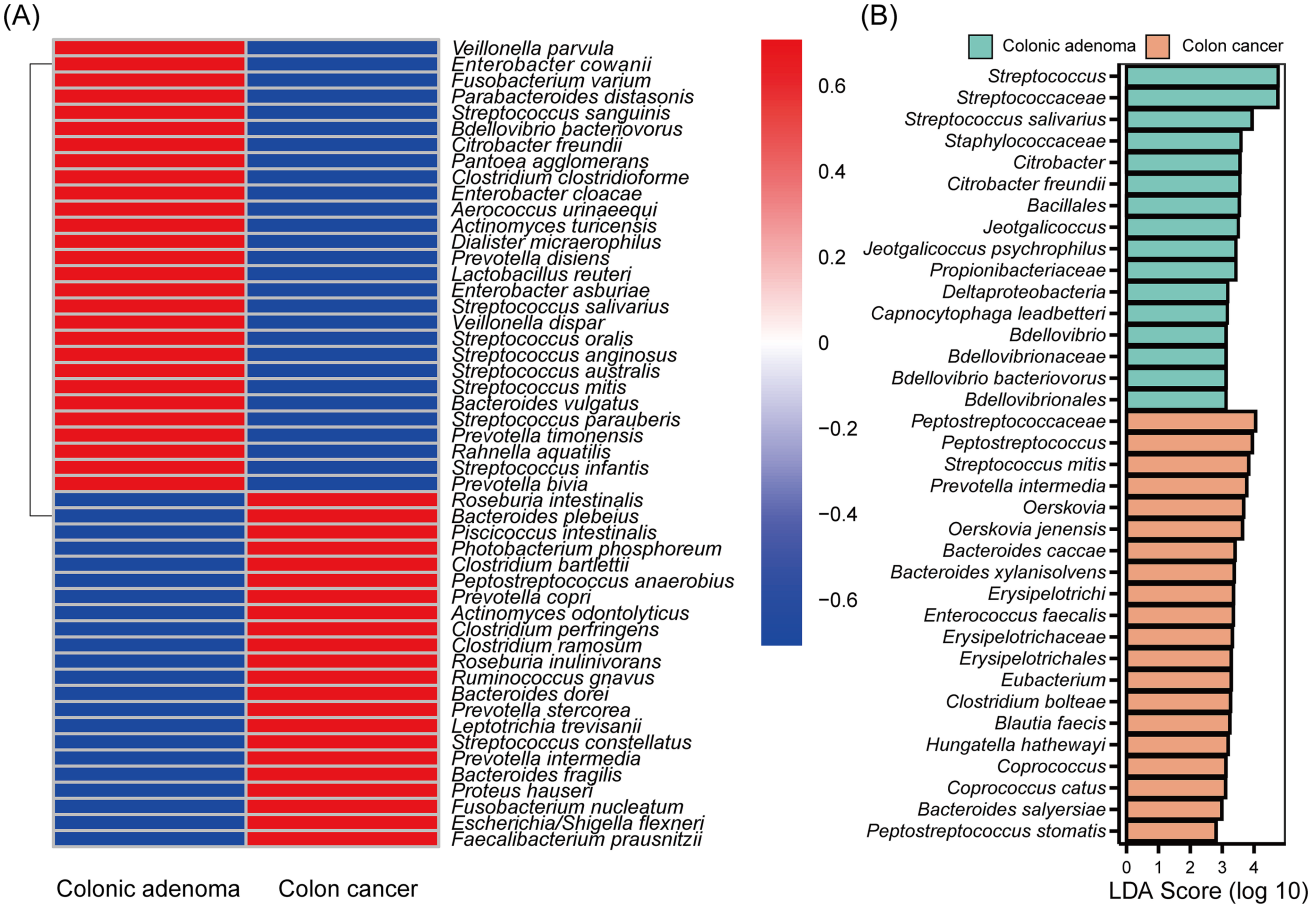
Identification of differentially abundant microbial taxa. Identification of differential microbial taxa between groups. **(A)** Clustering heatmap of species-level microbial abundance. Colour scale represents Z-score-transformed relative abundance (red: higher; blue: lower). The upper cluster was enriched in the adenoma group, including *Streptococcus* spp. (including *S. salivarius* and *S. australis*); the lower cluster was enriched in the cancer group, including *B. caccae*, *P. intermedia*, *F. nucleatum*, and *Escherichia/Shigella flexneri*. **(B)** LEfSe analysis (LDA score threshold > 3.0). Orange bars indicate taxa enriched in colon cancer; teal bars indicate taxa enriched in colonic adenoma.

Clustering heatmap analysis (Figure 4A) clearly reveals a “polarised” pattern in microbial abundance distribution between the two groups; in the clustering heatmap, sample clusters formed two main clusters. In the cluster enriched in the colon cancer group, the abundances of *F. nucleatum*, *P. intermedia* and *Escherichia/Shigella* were increased. In contrast, within the cluster enriched in the adenoma group, relative abundances of multiple *Streptococcus* species (including *S. salivarius* and *S. australis*) and *Prevotella bivia* were higher.

The results of the LEfSe analysis (LDA score threshold > 3.0) showed (Figure 4B) that *Peptostreptococcus stomatis* (LDA > 3.5) and *Prevotella intermedia* are the most characteristic differential taxa in the colon cancer group. In addition, *Bacteroides caccae*, *Bacteroides salyersiae*, *Coprococcus catus*, *Clostridium bolteae*, and *Enterococcus faecalis* were also clearly enriched. However, *F. nucleatum*, which appeared in the species clustering plot, did not reach the significance threshold in the LEfSe analysis. Compared with the colon cancer group, the taxonomic groups that were relatively enriched in the colonic adenoma group mainly belonged to the Streptococcaceae family, including *Streptococcus salivarius*. The genus *Peptostreptococcus* was also detected in the adenoma group, but its relative abundance was approximately one order of magnitude lower than in the colon cancer group.

### 3.7 Statistical validation of differential taxa and clinical correlations

**Figure 5:**
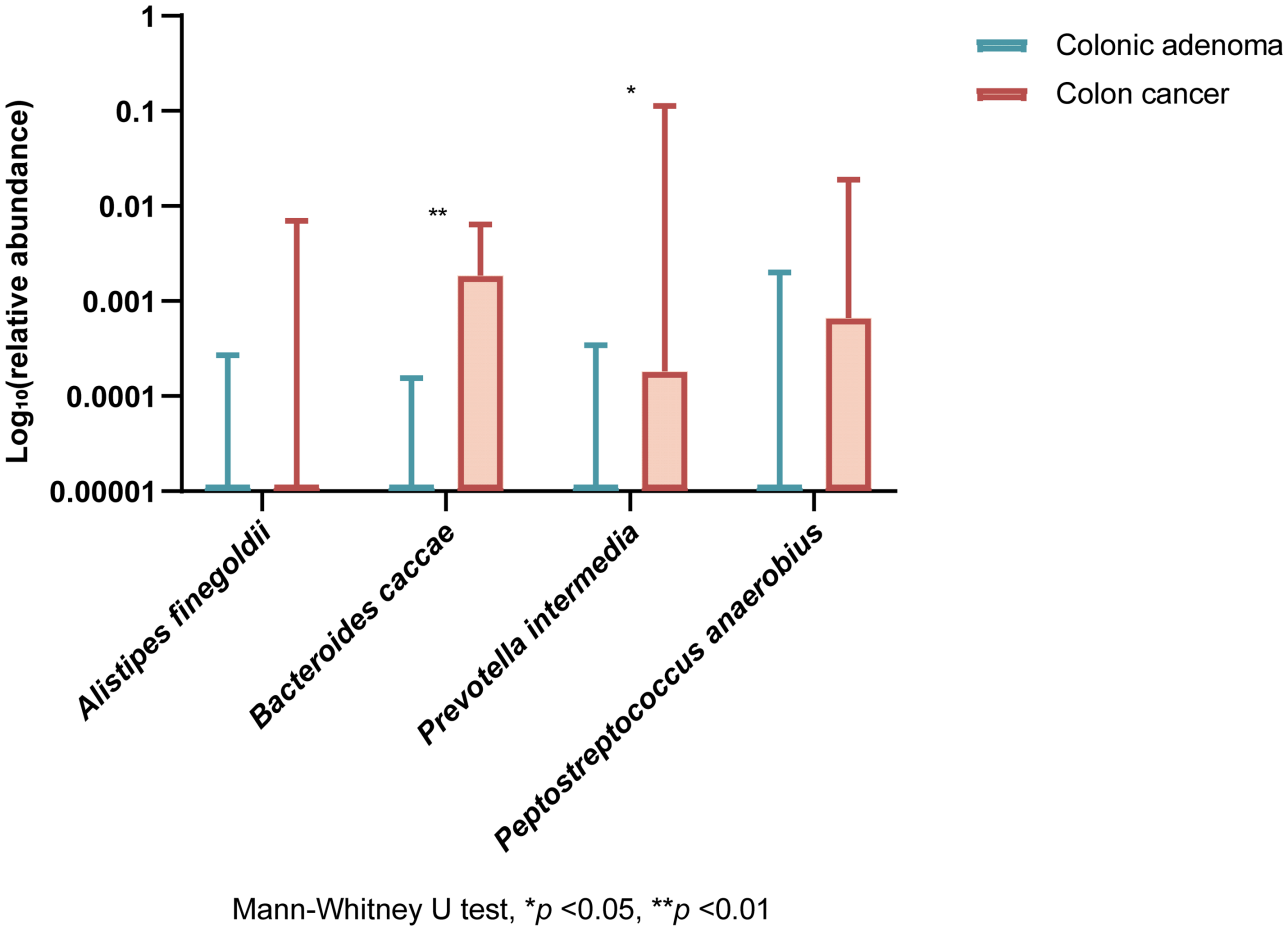
Multi-method validation of candidate differential taxa. Multi-method validation of candidate differential taxa. Relative abundance (Log₁₀-transformed) of four candidate taxa. Teal: colonic adenoma; orange: colon cancer. Mann–Whitney U test: *p < 0.05; **p < 0.01.

Multiple independent statistical validations were performed for candidate differential species. ANCOM-BC2 was employed to analyse intergroup differences, identifying four taxa with statistically significant differences between colon cancer and colonic adenoma (FDR-adjusted *p <* 0.05), including *A. finegoldii* (FDR-adjusted *p =* 0.00030), *B. caccae* (FDR-adjusted *p =* 0.00025), *P. intermedia* (FDR-adjusted *p* = 0.00347) and *Peptostreptococcus anaerobius* (FDR-adjusted *p* = 0.00031). LEfSe indicated an enrichment of *P. stomatis*, whereas ANCOM-BC2 identified *P. anaerobius*, a species within the same genus, as exhibiting more stable intergroup differences. The Mann–Whitney U test (Figure 5) and Fisher’s exact test (Table 3) were used to further confirm the relative abundance distributions of the two groups. The results showed that *B. caccae* (*p =* 0.004) and *P. intermedia* (*p =* 0.021) had higher relative abundances in the colon cancer group compared to the adenoma group.

**Table 3:** Detection rates and median abundance of four candidate differential taxa.

Bacterial species	Cases detected in colonic adenoma/25	Cases detected in colon cancer/25	Fisher *p*	Median abundance in the colonic adenoma group	Median abundance in the colon cancer group
*Alistipes finegoldii*	3	5	0.702	0.000179	0.003175
*Bacteroides caccae*	1	9	0.023	0.000150^a^	0.002488
*Prevotella intermedia*	1	7	0.049	0.000344^a^	0.002147
*Peptostreptococcus anaerobius*	4	6	0.725	0.001607	0.003874

Fisher’s exact test. *A. finegoldii* is included for display purposes despite non-significant Fisher’s *p* value, given its clinical relevance demonstrated by Spearman correlation analysis.

a Median reflects a single detectable case.

Fisher’s exact test revealed that the detection rate of *B. caccae* was higher in the colon cancer group (9/25) than in the adenoma group (1/25, *p =* 0.023), while *P. intermedia* also showed a similar trend (colon cancer group: 7/25 vs. adenoma group: 1/25, *p =* 0.049). *B. caccae* and *P. intermedia* were identified as differential species in both ANCOM-BC2 and LEfSe.

Firth’s penalised logistic regression (primary model, adjusted for sex) confirmed that *B. caccae* detection was significantly associated with cancer group membership (OR = 10.52, 95% CI: 2.02–110.08, *p* = 0.004), and *P. intermedia* detection was also significantly associated with cancer group membership in the sex-adjusted model (OR = 5.53, 95% CI: 1.04–56.04, *p* = 0.044). Full results for both taxa across both models are provided in Supplementary Table S1.

**Figure 6:**
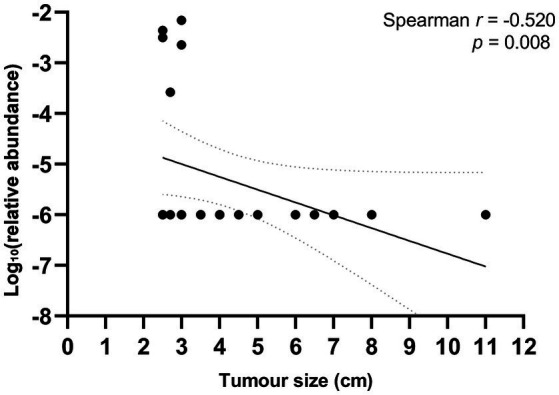
Correlation between *Alistipes finegoldii* abundance and tumour diameter. Spearman rank correlation was computed across all 25 colon cancer samples (*r* = −0.520, *p* = 0.008); 20 samples at the detection limit (relative abundance < 10^−6^; plotted at log₁₀ = −6) were assigned tied ranks, and 5 samples had detectable abundance. The solid line represents the linear regression fit for the 5 detectable samples; dashed lines indicate the 95% confidence interval.

Spearman’s rank correlation analysis was performed (Figure 6). In the colon cancer group, the relative abundance of *A. finegoldii* showed a negative correlation with tumour diameter across all colon cancer samples (*n* = 25; *r* = −0.520, *p* = 0.008; 20 samples at the detection limit were assigned tied ranks; only 5 samples had detectable abundance). The relative abundance of *B. caccae* showed a negative correlation with CA19-9 levels (Spearman’s *r* = −0.521, *p =* 0.022, *n* = 19).

As a descriptive exploratory tool, a Random Forest feature importance analysis (Supplementary Figure S2) suggested that *B. caccae* had the highest mean decrease in accuracy among candidate taxa; however, given the absence of formal cross-validation and the limited sample size, these results are presented solely as supplementary descriptive information and are not interpreted as classifier performance. As a pre-specified subgroup analysis, microbial community profiles were compared between left-sided (*n* = 31) and right-sided (*n* = 19) colon specimens; results were exploratory and are presented in full in Supplementary Figure S3.

## 4 Discussion

In this case–control study, we identified *B. caccae* and *P. intermedia* as consistently enriched in colon cancer tissue relative to colonic adenoma across four independent statistical methods, alongside a significant reduction in alpha diversity evenness in the cancer group. These findings add to a growing body of evidence that the tumour-associated microbiome undergoes taxon-specific compositional shifts during the adenoma-to-carcinoma transition (Feng et al., 2015; Nakatsu et al., 2015; Flemer et al., 2017), and demonstrate that such signals are detectable in archival FFPE tissue under a rigorous contamination control framework—a finding of practical relevance for retrospective cohort studies in which fresh-frozen tissue is unavailable.

The significant reductions in Shannon, Simpson and Pielou’s evenness indices in the colon cancer group are consistent with previous reports linking CRC progression to loss of microbial community evenness in tumour-associated and mucosal microbiome studies (Nakatsu et al., 2015; He et al., 2021). Notably, species richness indices (Chao1 and observed species) did not differ significantly between groups, indicating that the dominant change was a shift in community evenness rather than an outright loss of species. This pattern is consistent with the bacterial driver-passenger model, in which selective enrichment of specific taxa progressively restructures community composition as tumour conditions favour particular microorganisms (Tjalsma et al., 2012). In contrast to the taxon-level and evenness changes observed in alpha diversity, global community composition did not differ significantly between the adenoma and colon cancer groups (ANOSIM: *p* = 0.106; PERMANOVA: *p* = 0.137). This finding is consistent with observations from studies profiling the gut microbiome across stages of colorectal carcinogenesis, in which taxon-specific enrichment is detectable before any wholesale restructuring of community composition occurs (Nakatsu et al., 2015; Feng et al., 2015).

*B. caccae* showed the most consistent evidence of enrichment in the colon cancer group, identified across all four independent statistical methods (detection rate 9/25 vs. 1/25, Fisher’s *p* = 0.023). Flemer et al. (2017) reported enrichment of Bacteroides-affiliated taxa in CRC tumour tissue; the present study observed a comparable signal in FFPE-preserved material, suggesting this enrichment is stable across tissue preservation methods. *B. caccae* is a major producer of lipopolysaccharide (LPS) in the human gut; structurally distinct from classical LPS, Bacteroides-derived LPS has been shown to antagonise Toll-like receptor 4 (TLR4) signalling, potentially modulating local immune signalling (D'Hennezel et al., 2017; Vatanen et al., 2016). Bacteroides species additionally encode multiple polysaccharide utilisation loci with enzymatic capacity to degrade intestinal mucins (Martens et al., 2018), which may compromise epithelial barrier integrity. The broader oncogenic potential within this genus is illustrated by toxigenic *B. fragilis*, which disrupts the intestinal epithelial barrier via secreted toxins (Wu et al., 1998; Sears, 2009). Whether *B. caccae* exerts analogous pro-tumorigenic effects through these pathways warrants direct experimental validation.

In contrast to the statistically significant enrichment of *B. caccae*, another classic colorectal carcinogenesis-associated bacterium, *F. nucleatum*, another classic colorectal carcinogenesis-associated bacterium, did not meet the strict statistical threshold in this study. Although *F. nucleatum* showed a trend towards enrichment in the colon cancer group in species composition analysis and clustering heatmaps, it did not reach significance in either LEfSe or ANCOM-BC2 analyses. Of note, several Gram-negative taxa—including *F. nucleatum*, *Escherichia/Shigella flexneri*, *Proteus hauseri*, and *Leptotrichia trevisanii*—exhibited a visual enrichment trend in the hierarchical clustering heatmap of the colon cancer group, yet none achieved formal statistical significance. One plausible explanation is the susceptibility of thin-walled Gram-negative bacteria to DNA degradation and cross-linking during formalin fixation, reducing detection sensitivity compared with Gram-positive taxa—a phenomenon previously documented in FFPE microbiome studies (Cruz-Flores et al., 2022; Borgognone et al., 2021). Their measured abundances may therefore represent underestimates, and direct comparisons with fresh-tissue studies should be interpreted accordingly.

While the 5R short-fragment amplification strategy was specifically designed to mitigate amplification failures associated with FFPE-derived fragmented DNA (Fuks et al., 2018; Cruz-Flores et al., 2022), it does not eliminate all sources of taxon-differential detection bias inherent to the FFPE matrix. Several interpretive caveats therefore remain. First, formalin fixation induces progressive DNA fragmentation and cross-linking that reduces template quality in a taxon-dependent manner; Gram-negative organisms, whose thinner cell walls afford less protection against fixation-induced DNA damage, tend to show lower detection sensitivity in FFPE compared with fresh tissue, a pattern documented in matched FFPE–fresh comparisons of CRC microbiomes (Borgognone et al., 2021; Lam et al., 2021). This differential sensitivity likely contributes to the non-significant enrichment trends observed for *F. nucleatum* and *Escherichia/Shigella* in the present study, whose detection rates may underestimate their true biological prevalence. Second, because differential DNA preservation across taxa influences sequencing library composition, observed relative abundances reflect not only the underlying community structure but also variation in fixation-induced DNA integrity; direct quantitative comparisons with fresh-tissue abundance estimates should therefore be made with caution. Third, fixation duration, formalin concentration, and archival storage age varied across our retrospective specimen collection in a manner that could not be fully standardised (Cruz-Flores et al., 2022); this specimen-level technical heterogeneity may inflate within-group variability and reduce power for detecting smaller effect sizes, particularly for low-prevalence taxa. These considerations underscore the importance of prospective validation of the candidate taxa identified here in fresh or cryopreserved tissue cohorts.

*P. intermedia* was characteristically enriched in the colon cancer group across detection-rate and abundance metrics (7/25 vs. 1/25, Fisher’s *p* = 0.049; Mann–Whitney *p* = 0.021). Lo et al. (2022) reported *P. intermedia* enrichment in CRC tumour tissue, with synergistic effects alongside *F. nucleatum* in promoting adenoma malignant transformation; enrichment in faecal samples from a Vietnamese CRC cohort (Nhung et al., 2024) further supports a consistent distribution pattern across sample types and geographic populations. *P. intermedia* is traditionally classified as an oral opportunistic pathogen. The oral-gut migration hypothesis (Yang et al., 2019; Kitamoto et al., 2020) proposes that oral bacteria enter the digestive tract via swallowing and achieve ectopic colonisation under conditions of local microenvironmental acidification and aberrant mucin expression in tumour tissue (Koliarakis et al., 2019; Schmidt et al., 2019). These observations are consistent with the oral-gut migration hypothesis and suggest that *P. intermedia* may be associated with the colonic tumour microenvironment.

*A. finegoldii* presented a distinct pattern from the other two differential taxa. In ANCOM-BC2 analysis, *A. finegoldii* showed a significant abundance difference between groups (FDR-adjusted *p* = 0.00030), whereas Fisher’s exact test revealed no difference in detection rates (5/25 vs. 3/25, *p* = 0.702), indicating a distributional shift rather than a prevalence change. Of note, within the colon cancer group, *A. finegoldii* relative abundance showed a negative correlation with tumour diameter across all colon cancer samples (n = 25; *r* = −0.520, *p* = 0.008; 20 samples at detection limit included as tied ranks); only five samples had detectable abundance. This observation is strictly exploratory and no mechanistic interpretation is warranted at this stage. *Alistipes* species produce short-chain fatty acids with anti-inflammatory properties, and *A. finegoldii* has been reported to enhance the efficacy of immune checkpoint inhibitors in solid tumours (Parker et al., 2020; Wu et al., 2025). Prospective validation in larger cohorts is required before conclusion regarding a meaningful relationship between *A. finegoldii* abundance and tumour diameter can be drawn.

While this study has revealed differences in gut microbial taxa between patients with colonic adenomas and those with colon cancer, as well as associated clinical implications, there are still some limitations. First, the sample size was constrained by strict inclusion and exclusion criteria. *Post hoc* power analysis indicated adequate power for the primary differential abundance findings (*B. caccae*: 0.878; *P. intermedia*: 0.712), but the study was underpowered for detecting global community-level differences (PERMANOVA *R*^2^ = 0.024), and the sparse outcome for *P. intermedia* (8/50 positive cases) necessitated Firth’s penalised regression; primary inference for this taxon therefore relies on ANCOM-BC2 and Fisher’s exact test. All findings should be regarded as preliminary and require replication in larger cohorts. Second, contamination removal relied on a pre-specified conservative prevalence threshold rather than a probabilistic decontamination algorithm applied at the pipeline design stage; this conservative approach may have led to over-removal of some taxa relative to a statistical decontamination criterion, though retrospective decontam analysis confirmed that none of the four differentially abundant taxa were affected by this over-conservatism. Third, the retrospective cross-sectional design precludes causal inference; prospective longitudinal studies with mechanistic *in vitro* and *in vivo* validation are needed to clarify the specific roles of *B. caccae*, *P. intermedia*, and *A. finegoldii* in the adenoma-to-carcinoma transition. Fourth, the volumetric difference between surgical resection specimens and endoscopic resection represents a methodological challenge for FFPE microbiome studies. To improve inter-specimen comparability, future work could explore density-standardised protocols; for example, a standard micro-tissue core punch could be used to extract a defined volume from the macroscopic tumour block prior to DNA extraction, thereby homogenising fixation density and avoiding tissue-volume-mediated false negatives in validation cohorts. Finally, future studies with larger, unmatched cohorts should conduct formal redundancy analysis (RDA) or linear mixed-effect models to rigorously assess the contribution of clinical comorbidities as potential confounders.

## 5 Conclusion

This study demonstrates the feasibility of tumour-associated microbiome profiling in archival FFPE tissue and identifies candidate compositional signatures of the colonic adenoma-to-carcinoma transition, including *B. caccae* and *P. intermedia* as consistently enriched taxa warranting prospective validation as potential biomarkers of colonic adenoma-to-carcinoma transition. The exploratory negative correlation between *A. finegoldii* abundance and tumour diameter may reflect microbiome composition changes during tumour progression, though validation in larger cohorts is required. These findings provide a preliminary evidence base supporting the integration of FFPE-based tumour microbiome profiling into future retrospective and prospective cohort studies, with mechanistic investigation of candidate taxa as a priority for subsequent research.

## Data Availability

The raw sequencing data (FASTQ files for all 50 tissue samples and 4 sampling negative controls, *n* = 54) are deposited in the NCBI Sequence Read Archive (SRA) under BioProject accession number PRJNA1476454 (https://www.ncbi.nlm.nih.gov/bioproject/PRJNA1476454). The raw sequencing data for the 5 DNA extraction negative controls and 5 PCR amplification negative controls (*n* = 10), which were processed by the commercial sequencing provider (Personal Biotechnology Co., Ltd., Shanghai, China) as part of their standard quality pipeline, are available upon reasonable request to the corresponding authors.
