# Supplementary material for: Tumour tissue-associated microbiome differences between colonic adenoma and carcinoma revealed by 5R 16S rRNA sequencing of formalin-fixed paraffin-embedded tissues: a case–control study
Source: Front Microbiol. 2026 Jul 10;17:1880194. doi: 10.3389/fmicb.2026.1880194 (PMC13396170; doi:10.3389/fmicb.2026.1880194)
Supplement: Supplementary file 1 [file Supplementary_file_1.zip › Supplementary Table_S1.pdf]

Supplementary Material

Supplementary Table S1. Binary logistic regression: covariates associated with *B. caccae* and *P. intermedia* detection.

Variable	OR	95% CI (profile likelihood)	*p*	EPV
Panel A: *B. caccae* (10 positive cases / 50 total)				
Model 1 — adjusted for sex (primary model; EPV = 5.0)				
Group (cancer vs. adenoma)	**10.52**	**2.02 – 110.08**	**0.004**	5.0
Sex (male vs. female)	0.52	0.10 – 2.56	0.419	
Model 2 — additionally adjusted for age, BMI (sensitivity; EPV = 2.5)				
Group (cancer vs. adenoma)	**10.87**	**1.88 – 125.56**	**0.006**	2.5
Sex	0.50	0.10 – 2.49	0.396	
Age (per year)	1.00	0.94 – 1.06	0.855	
BMI (per unit)	1.06	0.81 – 1.44	0.694	
Panel B: *P. intermedia* (8 positive cases / 50 total)				
Model 1 — adjusted for sex (primary model; EPV = 4.0)				
Group (cancer vs. adenoma)	**5.53**	**1.04 – 56.04**	**0.044**	4.0
Sex (male vs. female)	2.92	0.52 – 30.40	0.239	
Model 2 — additionally adjusted for age, BMI (sensitivity; EPV = 2.0)				
Group (cancer vs. adenoma)	3.96	0.69 – 40.38	0.127	2.0
Sex	3.38	0.56 – 37.26	0.194	
Age (per year)	1.00	0.95 – 1.07	0.900	
BMI (per unit)	0.89	0.67 – 1.20	0.409	

Supplementary Table S1 note: OR, odds ratio; CI, confidence interval (profile likelihood intervals, as produced by Firth's penalized maximum likelihood estimation); EPV, events per variable. Dependent variable: taxon detection (present = 1, absent = 0), $n = 50$. Method: Firth's penalized MLE (Heinze and Schemper, 2002). The Model 2 result for *P. intermedia* (EPV = 2.0) is presented for completeness but should be interpreted with caution; the sex-adjusted Model 1 is the primary model for this taxon. Bold rows indicate the primary predictor of interest.

Supplementary Table S2. Per-sample sequencing quality metrics ($n = 50$).

Supplementary Table S3. Complete list of 1,058 ASVs removed by Stage 3 prevalence filtering.

Supplementary Table S4. 35 ASVs flagged by retrospective decontam analysis.

Supplementary Figure S1. Rarefaction curves for all samples.

Supplementary Figure S1 note: Group-averaged rarefaction curves for colonic adenoma and colon cancer groups, confirming adequate sequencing coverage for both groups.

Supplementary Figure S2. Random Forest feature importance analysis.

Supplementary Figure S2 note: Left panel: Z-score-transformed relative abundance heatmap for candidate taxa across all samples (B001–B025, colonic adenoma group; C001–C025, colon cancer group); colour scale reflects Z-score values. Right panel: MDA derived from the Random Forest model, reflecting the relative classification contribution of each taxon. These results are presented solely as supplementary descriptive information; no formal cross-validation was performed and no classifier performance metrics are reported.

Supplementary Figure S3. Subgroup analysis of left- and right-sided colon microbiota.

Supplementary Figure S3 note: Subgroup analysis of left- and right-sided colon microbiota. Linear discriminant analysis (LDA) bar chart comparing microbial communities between left-sided colon specimens (sigmoid colon and descending colon, $n = 31$) and right-sided colon specimens (ascending colon and transverse colon, $n = 19$). LDA score threshold > 3.0. LEfSe analysis identified Bacteroidaceae and Lachnospiraceae as enriched in right-sided specimens, whilst left-sided specimens showed relative enrichment of Prevotellaceae. Beta diversity analysis (ANOSIM: $R = 0.054$, $p = 0.106$; 999 permutations; PERMANOVA: $R^2 = 0.024$, $p = 0.126$; 999 permutations, Bray-Curtis dissimilarity) indicated no statistically significant difference in overall community structure between anatomical subgroups. Given the limited sample sizes within each subgroup, all findings should be regarded as exploratory and require replication in adequately powered cohorts.
