# Supplementary figures and images for: Tumour tissue-associated microbiome differences between colonic adenoma and carcinoma revealed by 5R 16S rRNA sequencing of formalin-fixed paraffin-embedded tissues: a case–control study
Source: Front Microbiol. 2026 Jul 10;17:1880194. doi: 10.3389/fmicb.2026.1880194 (PMC13396170; doi:10.3389/fmicb.2026.1880194)

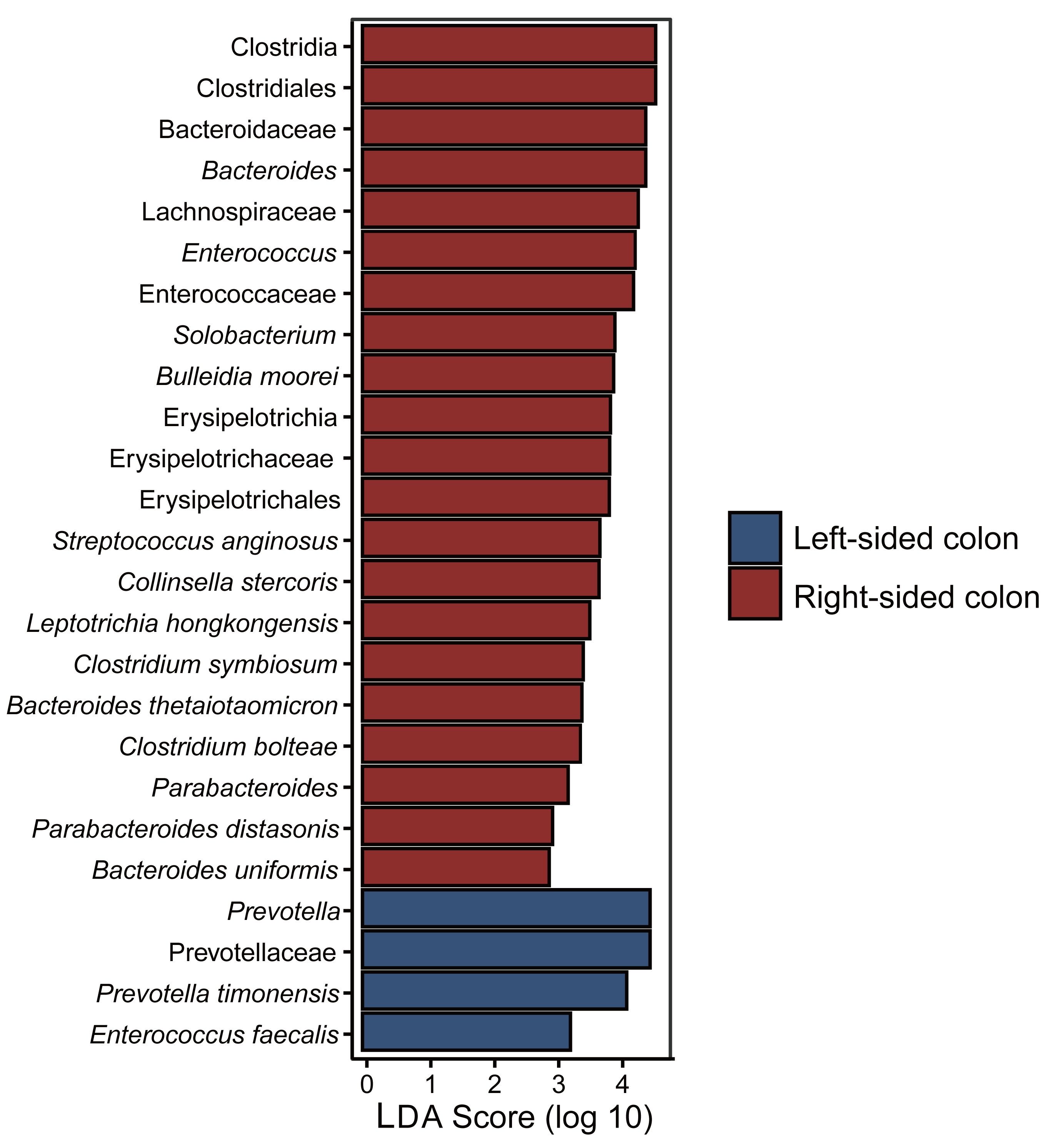

Supplement: Supplementary file 1 [file Supplementary_file_1.zip › Supplementary Figure S3.TIF]

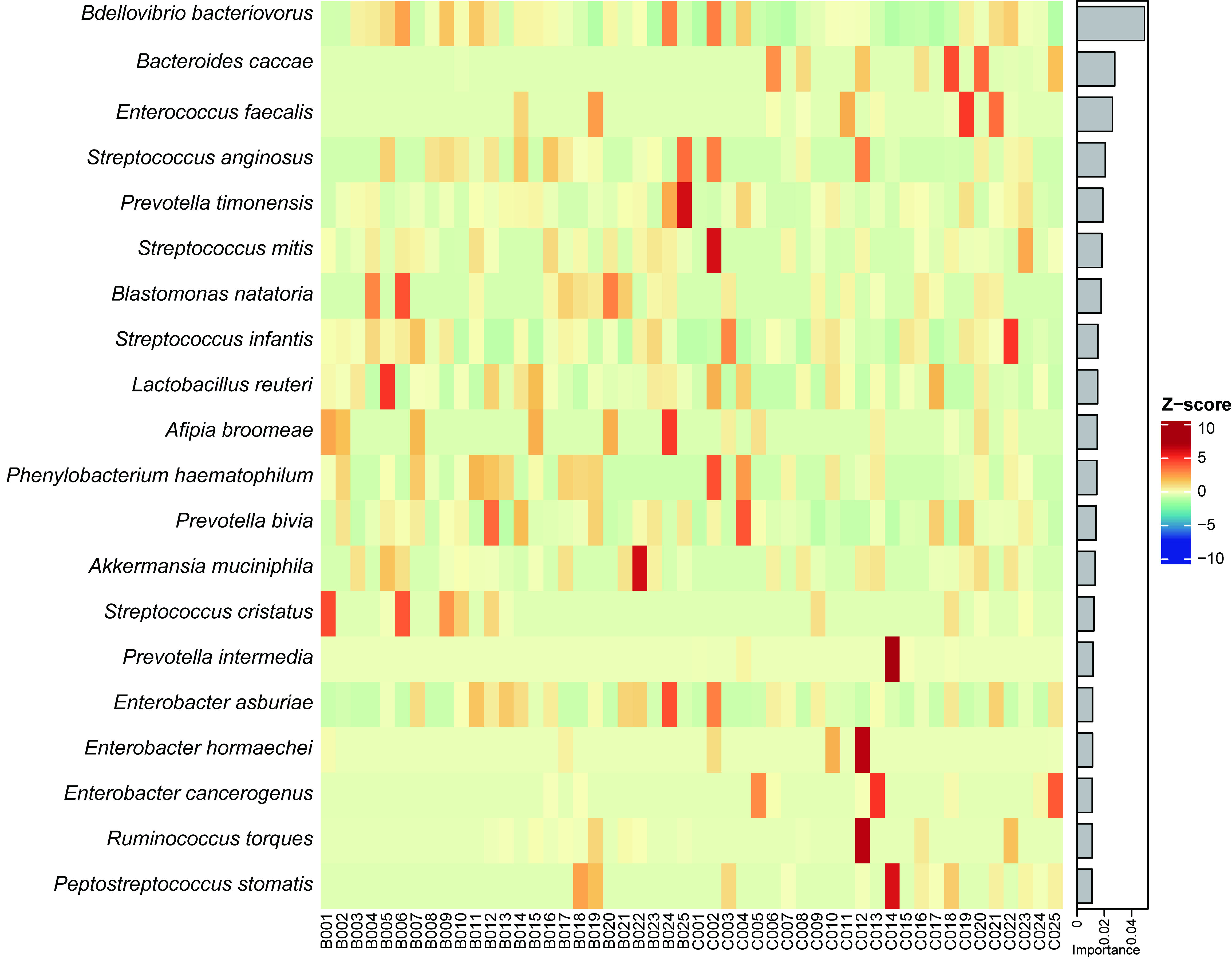

Supplement: Supplementary file 1 [file Supplementary_file_1.zip › Supplementary Figure S2.TIF]

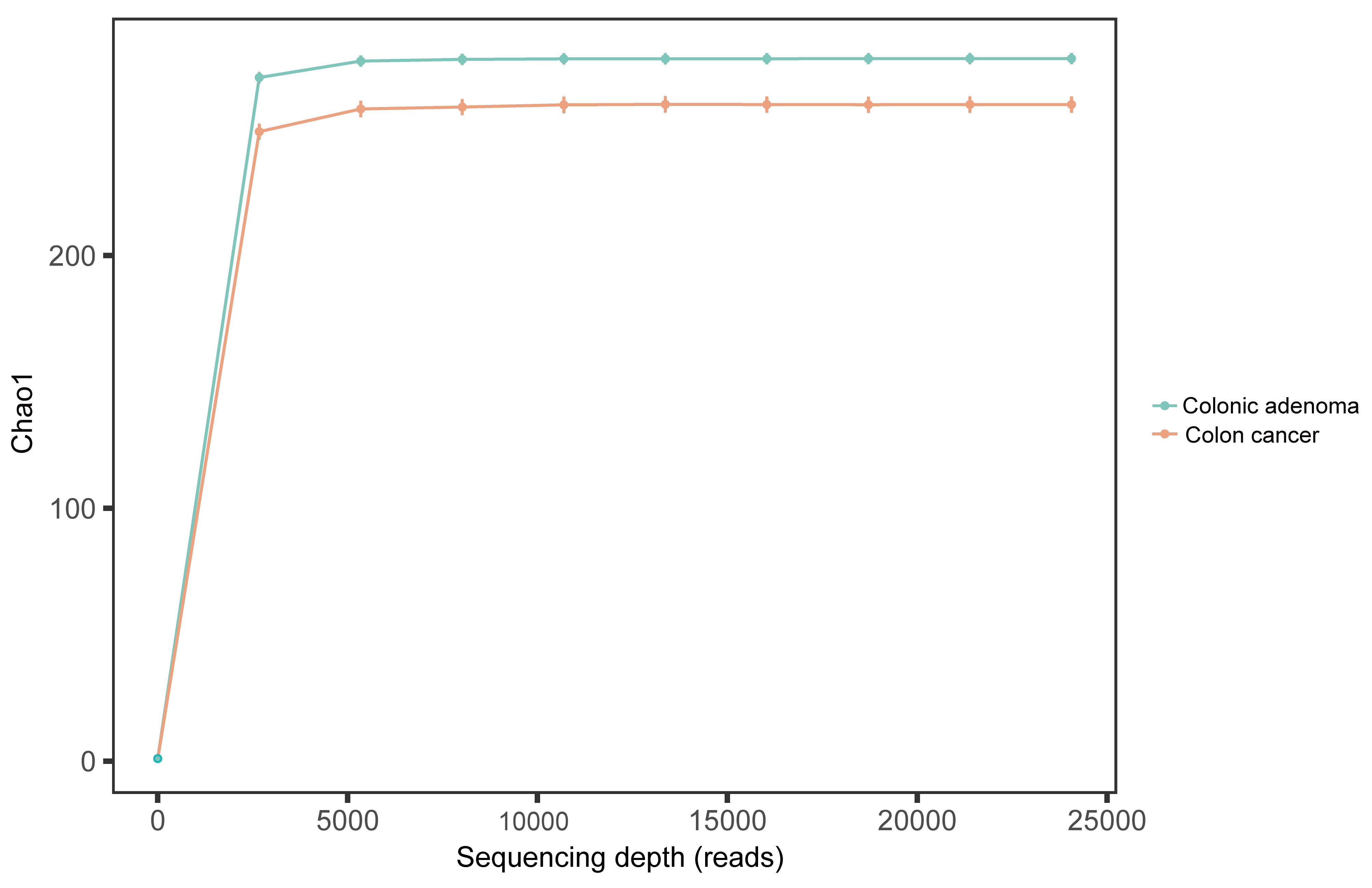

Supplement: Supplementary file 1 [file Supplementary_file_1.zip › Supplementary Figure S1.TIF]
